# Supplementary material for: Analysis of the genomic architecture of a complex trait locus in hypertensive rat models links Tmem63c to kidney damage
Source: eLife. 2019 Mar 22;8:e42068. doi: 10.7554/eLife.42068 (PMC6478434; doi:10.7554/eLife.42068)
Supplement: Figure 3—source data 1. [file elife-42068-fig3-data1.docx]

**Figure 3 – source data 1. Predicted effects of variants identified by NGS in the candidate region on rat chromosome 6 in human**

| **Gene** | **Variant Position** | **Amino acid exchange** | | **Effect of sequence variant** | | **PROVEAN**  **Score** | | **PhastCons Score** |
| --- | --- | --- | --- | --- | --- | --- | --- | --- |
|  | **RAT** | **HUMAN** | | **HUMAN** | | **HUMAN** | |  |
|  |  |  |  | |  | |  |  |
| *Acot4* | 107,518,131 | Gly → Ser | | non-synonymous | | -4.000 | | 0.82 |
| *Acot5** | 107,551,446 | Arg → Ser | | non-synonymous | | -2.880 | | 0.74 |
|  | 107,551,528 | Arg → His | | non-synonymous | | -2.670 | | 0.97 |
|  | 107,551,717 | Pro → Arg | | **Stop codon gained** | | -5.140 | | 0.52 |
|  | 107,557,092 | Leu → Stop | | non-synonymous | | NA | | 0.29 |
| *Acot6* | 107,590,006 | Leu → Pro | | non-synonymous | | -5.520 | | 0.01 |
| *Ptgr2* | 108,029,833 | Cys → Arg | | non-synonymous | | 3.570 | | 0.93 |
| *Ngb* | 111,128,730 | NA | | **synonymous** | | 0 | | 0.97 |
|  | 111,131,291 | NA | | **UTR5’ deletion** | | NA | | 0.41 |

*the human homologue is *ACOT2*
