# Supplementary material for: Analysis of the genomic architecture of a complex trait locus in hypertensive rat models links Tmem63c to kidney damage
Source: eLife. 2019 Mar 22;8:e42068. doi: 10.7554/eLife.42068 (PMC6478434; doi:10.7554/eLife.42068)
Supplement: Figure 4—source data 2. [file elife-42068-fig4-data2.docx]

**Figure 4 – source data 2. Primer list for quantitative real-time PCR analysis.**

| **Gene** | | **GenBank**  **accession no.** | | **Sense Primer**  **5’ → 3’** | | **Antisense Primer**  **5’ → 3’** | **Amplicon**  **length (bp)** | | **Exon** | **Primer design by** | |  |
| --- | --- | --- | --- | --- | --- | --- | --- | --- | --- | --- | --- | --- |
|  | |  | |  | |  |  | |  |  | |  |
| *Acot3* | NM_001108041 | | CCCAAGGACGTGGACATTAT | | ACCCCAACTCCTGAACCTTT | | 95 | 2 + 3 | | | Primer3 | |
| *Acot5* | NM_001079709 | | TCCTGCCTCCAGGAACTG | | ATTCCAGAAGTCCACCTCCA | | 72 | 1/2 + 2 | | | Primer3 | |
| *Esrrb* | NM_001008516 | | CAGCTCGTACGCAGGTACA | | CGCTGGCTCAGCTCATAGT | | 170 | 5 + 6 | | | OLIGO | |
| *Flvcr2* | NM_199109 | | TACCACATCAGCATCATGTTCTAC | | CCCTGCTGGGAGGGTACT | | 106 | 2 + 3 | | | OLIGO | |
| *Hmbs* | NM_013168 | | TgAAAACCTTgTACCCTggCATA | | TCCAATCTTAgAgAgTgCAgTATCAAgA | | 92 | 3 + 4/5 | | | PrExpr | |
| *Jdp2* | NM_053894 | | GAGTTCCTGCAGAGGGAGTC | | CGTCCTGACGCTGTCTGTAC | | 153 | 3/4 + 4 | | | OLIGO | |
| *Papln* | XM_006225840 | | GTGTGTGGTGGCTGGAGAC | | TGGATCAACAGTGTGCCATC | | 111 | 25 + 26 | | | Primer3 | |
| *Rps6kl1* | XM_006225853 | | TGTTGCGATACTTCGTGAGC | | TGAAAGTGGTCCTGGGAGAG | | 100 | 5 + 6 | | | Primer3 | |
| *Tgfb3* | NM_013174 | | TCAGGCCCTTGCCCTTAC | | GGGGGTTCTGCCAACATAG | | 156 | 6 + 7 | | | OLIGO | |
| *Tmem63c* | NM_001108045 | | TCAACACCATCGACATGTACA | | TAGGAAAGCGGAGAAGTAGAC | | 140 | 16 + 17 | | | OLIGO | |
| *Vash1* | XM_001058397 | | GCCACCTGGGAAAGGATGT | | CGTTGTCGGCTGGAAAGTAG | | 135 | 1 + 2 | | | OLIGO | |

Primers (TIB MOLBIOL GmbH) were designed by Primer3web (Primer 3) (version 4.0.0) ([Koressaar & Remm, 2007](#_ENREF_32); [Untergasser et al., 2012](#_ENREF_65)), PrimerExpress (PrExpr) (version 2.0.0, Applied Biosystems, Darmstadt, Germany) and OLIGO (version 4.1, published by National Biosciences, Inc 3650 Annapolis Lane Plymouth, MN 55447, USA).
